# Iodothyronine deiodinase enzyme activities in bone

**DOI:** 10.1016/j.bone.2008.03.019

**Published:** 2008-07

**Authors:** Allan J. Williams, Helen Robson, Monique H.A. Kester, Johannes P.T.M. van Leeuwen, Stephen M. Shalet, Theo J. Visser, Graham R. Williams

**Affiliations:** a Molecular Endocrinology Group, Division of Medicine and Medical Research Council (MRC) Clinical Sciences Centre, Imperial College London, Hammersmith Hospital, London W12 0NN, UK; b Department of Clinical Research, Christie Hospital National Health Service (NHS) Trust, Manchester, M20 4BX, UK; c Cancer Tissue Bank Research Centre, Department of Pathology, Duncan Building, University of Liverpool, Daulby Street, L69 3GA, UK; d Department of Internal Medicine, Erasmus University Medical Center, 3015 GE Rotterdam, The Netherlands; e Department of Endocrinology, Christie Hospital NHS Trust, Manchester, M20 4BX, UK

**Keywords:** Thyroid hormone metabolism, MCT8, Chondrocyte, Osteoblast, Osteoclast

## Abstract

Euthyroid status is essential for normal skeletal development and maintenance of the adult skeleton, but the mechanisms which control supply of thyroid hormone to bone cells are poorly understood. Thyroid hormones enter target cells via monocarboxylate transporter-8 (MCT8), which provides a functional link between thyroid hormone uptake and metabolism in the regulation of T3-action but has not been investigated in bone. Most circulating active thyroid hormone (T3) is derived from outer ring deiodination of thyroxine (T4) mediated by the type 1 deiodinase enzyme (D1). The D2 isozyme regulates intra-cellular T3 supply and determines saturation of the nuclear T3-receptor (TR), whereas a third enzyme (D3) inactivates T4 and T3 to prevent hormone availability and reduce TR-saturation. The aim of this study was to determine whether MCT8 is expressed in the skeleton and whether chondrocytes, osteoblasts and osteoclasts express functional deiodinases. Gene expression was analyzed by RT-PCR and D1, D2 and D3 function by sensitive and highly specific determination of enzyme activities. MCT8 mRNA was expressed in chondrocytes, osteoblasts and osteoclasts at all stages of cell differentiation. D1 activity was undetectable in all cell types, D2 activity was only present in mature osteoblasts whereas D3 activity was evident throughout chondrocyte, osteoblast and osteoclast differentiation in primary cell cultures. These data suggest that T3 availability especially during skeletal development may be limited by D3-mediated catabolism rather than by MCT8 mediated cellular uptake or D2-dependent T3 production.

## Introduction

Bone development occurs via endochondral ossification, in which proliferation, differentiation and apoptosis of growth plate chondrocytes is co-ordinated to produce a cartilage scaffold that is mineralized during bone formation [39]. Hypothyroidism in children results in delayed bone age and growth arrest [58], and T4-treatment induces rapid “catch-up” growth [60]. Juvenile thyrotoxicosis, by contrast, advances bone age and accelerates growth but results in short stature due to premature closure of the growth plates [68]. In adults, hypothyroidism reduces bone turnover, whereas thyroid hormone excess increases bone formation and resorption [52]. The increased and unbalanced bone turnover in thyrotoxicosis generates a net increase in osteoclast activity leading to accelerated bone loss, osteoporosis and increased fracture susceptibility [9,53,75]. Thus, euthyroidism is critical for skeletal development and maintenance of adult bone [7,32].

The iodothyronine deiodinases are selenoenzymes that activate or inactivate thyroid hormones. The type 2 deiodinase (D2) catalyzes removal of an outer ring iodine atom from the prohormone thyroxine (T4) with an apparent Michaelis constant (*K*_m_) of 10^− 9^ M to generate the active product 3,5,3'-triiodothyronine (T3). By contrast, the D3 isozyme inactivates T3, or prevents T4 from being activated, by catalyzing the removal of an inner ring iodine with a *K*_m_ of 10^− 9^ M to generate 3,3'-diiodothyronine (T2) or 3,3',5'-triiodothyronine (reverse T3, rT3) respectively. The D1 enzyme however is inefficient, with a *K*_m_ of 10^− 6^–10^− 7^ M, and catalyzes removal of inner or outer ring iodine atoms in equimolar proportions to generate T3, rT3 or T2 depending on the substrate. Most circulating T3 is derived from T4 by the actions of D1 in liver and kidney, although D2 in skeletal muscle also contributes [12,14,47]. Nevertheless, the primary action of D2 is to determine the intra-cellular concentration of T3 and level of saturation of the nuclear T3-receptor (TR). Its lower *K*_m_ enables efficient local generation of T3 at times of T4 deprivation and D2 is thought to protect vital structures from periods of hypothyroidism. Thyroid hormones enter target cells via monocarboxylate transporter-8 (MCT8) [28] and other transporter proteins [36]. MCT8 is expressed in many T3-responsive tissues, but has not been investigated in bone [36]. T4 treatment of cells co-expressing MCT8 and D2 results in increased T3 production and hormone responsiveness [28], demonstrating a functional link between thyroid hormone uptake and metabolism in the regulation of T3-action.

The inactivating D3 isozyme prevents thyroid hormone access to specific tissues at critical times and reduces TR-saturation [12,14]. D3 is expressed in fetal tissues and placenta where it prevents maternal thyroid hormone access to the developing fetus [77]. At this time unoccupied TRs are critical factors that generally maintain cell proliferation and prevent differentiation [19,27]. The sharp rise in T3 availability at birth in mammals is analogous to the T3-dependent metamorphosis climax in amphibians [35] and a similar period at hatching in birds [73], and depends on tightly regulated temporo-spatial expression of D2 and D3 [8]. Increased pituitary D2 expression correlates with maturation of the hypothalamic–pituitary–thyroid (HPT) axis whilst its expression in T3-target tissues, concomitant with reduced expression of D3, results in conversion of unoccupied TRs into occupied TRs and the initiation of cell differentiation [18,27,35,46,54,62]. Thus, the TR acts as a deiodinase-dependent developmental switch that regulates maturation of T3-dependent tissues. Accordingly, a role for D2 to regulate ossification in the developing embryo has emerged [23]. In this case Hedgehog signalling in the perichondrium activates the ubiquitin ligase subunit WSB-1 leading to degradation of D2 protein in the growth plate and increased PTHrP signalling, which regulates the pace of chondrocyte differentiation during early skeletogenesis [23].

Current information regarding expression of the deiodinases in bone is inconsistent. There are no previous studies investigating MCT8 or D3 in skeletal cells and analysis of D1 is incomplete. A single study in chondrogenic ATDC5 embryonal carcinoma cells was discordant, demonstrating detectable D1 mRNA expression and an absence of D1 enzyme activity [50], but D1 has not been investigated in primary chondrocytes. Several biochemical studies have also failed to identify 6-*n*-propylthiouracil (PTU) sensitive D1 activity in osteoblastic cells [24,30,42], suggesting D1 may be absent from osteoblasts. D1 expression has not been studied in osteoclasts. Expression of D2 has also never been investigated in osteoclasts but previous studies in chondrocytes and osteoblasts are contradictory. D2 mRNA has been demonstrated in chicken growth plate chondrocytes [69] and in ATDC5 cells, mouse calvarial osteoblasts and bone marrow stromal ST2 cells [50]. Some studies comparing relative potencies of T3 and T4 and using the D1 inhibitor PTU have suggested indirectly that D2 activity may also be present in primary chondrocytes, ATDC5 cells and a whole organ bone resorption assay [1,17,50], whereas other studies of growth plate organ cultures and primary chondrocytes reached the opposite conclusion [2,16,76]. Biochemical studies using radiolabeled rT3 substrate identified a D2-like activity in ATDC5 cell extracts and growth plate organ culture extracts by measurement of ^125^I^−^ release following assay incubation periods of 6–12 h [50]. In these experiments characterization of D2-like activity was incomplete and the 5'-deiodination detected has been proposed to be non-specific [30]. There are further inconsistencies regarding D2 expression in osteoblastic cells. D2-like 5'-deiodination activity was undetectable in UMR106 osteosarcoma cells [42] and human fetal osteoblasts [24], whereas Gouveia et al. [30] reported low levels of well-characterized specific D2 activity in osteoblastic cells as well as substantial non-specific 5'-deiodination of undefined origin. In addition to these studies, Morimura et al. [51] reported D2-like activity in SaOS-2 osteosarcoma cells and human NHOst cells. However, similar to studies in ATDC5 cells [50], detection of D2-like activity was based solely on the release of ^125^I^−^ following incubation of cell extracts with radiolabelled T4 or rT3 [51]. As release of ^125^I^−^ may result from non-specific degradation of substrate [30], the specificity of D2-like activity identified in osteoblastic cells [51] requires clarification.

The aim of this study was to determine whether MCT8 and D3 are expressed in bone and clarify expression of D1 and D2 in skeletal cells. We studied chondrocytes, osteoblasts and osteoclasts comprehensively and determined deiodinase activities using highly specific and sensitive assays that determined production of iodothyronine metabolites directly by high performance liquid chromatography (HPLC).

## Materials and methods

### Chondrocytes — ATDC5 cells and primary growth plate chondrocytes

ATDC5 cells were maintained in DMEM/Ham's F12 (Life Technologies, Paisley, UK) containing 5% fetal calf serum (FCS), 10 μg/ml transferrin (Sigma, Poole, UK) and sodium selenite 100 nM [3,70]. Cells were plated (12,500 cells/cm^2^) in medium containing 5% FCS stripped of thyroid hormones (SFCS) [64]. On day 1, medium was replaced with medium containing 5% SFCS and 10 μg/ml bovine insulin ± T3 (10 nM) or T4 (10 or 100 nM) in the absence or presence of PTU (0.1 or 1 mM). Growth was measured at days 3, 7 and 10 using 0.4% sulforhodamine-B (SRB; Sigma) as an indicator of cell number [61,70]. We previously reported that alkaline phosphatase (ALP) expression is accelerated by T3 in ATDC5 cells as they undergo differentiation [70]. Cells were stained for ALP using 5-bromo-4-chloro-3indolylphosphate/nitroblue tetrazolium as substrate (BCIP/NBT; Sigma). To investigate deiodinase enzyme activities, cells were treated with dexamethasone 1 µM, T3 100 nM, T4 100 nM or dibutyryl cAMP 1 mM for 24 h, or phorbol-12-myristate-13-acetate (TPA) 0.1 µM for 6 h prior to harvest. Proximal tibia growth plate chondrocytes were isolated from 3, 6 and 12 week male rats as described [61]. Cells were washed three times in serum-free medium (SFM) and diluted in DMEM/F12 supplemented with 10% FCS (Harlan SeraLab, Loughborough, UK), 10 μg/ml transferrin (Sigma) and sodium selenite 100 nM, before seeding at 7500–12,500 cells/cm^2^ [61]. Primary cells were cultured until 75% confluent (4–6 days), and treated with dexamethasone 1 µM, T3 100 nM, T4 100 nM or dibutyryl cAMP 1 mM for 24 h, or TPA 0.1 µM for 6 h.

### Osteoblasts — MC3T3-E1 cells, rat and mouse neonatal calvarial osteoblasts and SV-HFO cells

Neonatal calvarial osteoblasts were isolated by sequential collagenase digestion [10,71]. Primary osteoblasts and mouse osteoblastic MC3T3-E1 cells (ATCC CRL-2593) were seeded at 15,000 cells/cm^2^ and maintained for up to 28 days in α-MEM plus 10% SFCS, 50 μg/ml ascorbic acid, β-glycerophosphate 2 mM, sodium selenite 100 nM, penicillin/streptomycin 18 U/ml and amphotericin B 1.35 μg/ml at 37 °C and 5% CO_2_. Calvarial osteoblasts and MC3T3-E1 cells were treated with T3 100 nM, T4 100 nM or dexamethasone 1 µM throughout the period of culture. SV-HFO human fetal osteoblasts [20] were seeded at 10,000 cells/cm^2^ and maintained in MEM (Gibco BRL, Paisley, UK) plus 20 mM HEPES, 100 IU/ml penicillin, 100 µg/ml streptomycin, 1.8 mM CaCl_2_"2H_2_O, 100 nM sodium selenite and 2% SFCS at 37 °C and 5% CO_2_ [25]. SV-HFO cells were treated with MEM alone or with medium supplemented with T3 100 nM or dexamethasone 1 µM for 1, 7, 14 or 21 days.

### Osteoclasts — primary rat and mouse osteoclasts and RAW 264.7 cells

Bone marrow was obtained from tibias and femurs of 6–8 week old male rats or mice and cultures established in α-MEM supplemented with 10% SFCS, 100 nM sodium selenite, hM-CSF 25 ng/ml and RANKL 10 ng/ml at 37 °C in 5% CO_2_ [41]. The following day non-adherent cells were removed, filtered with a 70 μm strainer and plated at 150,000 cells/cm^2^ in α-MEM plus 10%, hM-CSF 25 ng/ml, 1% glutamine and penicillin/streptomycin 18 U/ml in the absence or presence of RANKL 10 ng/ml. After 5 days, cells were stained with tartrate-resistant acid phosphatase (TRAP) and examined for formation of multinucleated osteoclasts, which occurred in all cultures in the presence of RANKL. Murine RAW 264.7 monocytic cells (ATCC TIB-71) were cultured in DMEM containing 10% SFCS and sodium selenite 100 nM in the absence or presence of RANKL 20 ng/ml (R&D Systems, Abingdon, UK) [43,74].

### Measurement of deiodinase activities

Experiments to determine deiodinase activities in skeletal tissues and primary cells required large numbers of animals to obtain sufficient tissue sample for analysis, and assays were optimized to ensure that deiodinase activities were measured reliably and reproducibly. Enzyme activities were determined using highly specific and sensitive assays that measured ^125^I^−^ release as well as direct determination of iodothyronine metabolite production by HPLC [29]. In some instances cells were treated with dexamethasone, cAMP or TPA to induce deiodinase activities above basal levels in order to facilitate detection of enzyme activity under optimal conditions and determine whether the deiodinases are regulated by extra-cellular signals in skeletal cells [14]. Treatment of cells with dexamethasone [72] or cAMP [44] induces D2 whereas treatment with TPA induces D3 [22], thereby resulting in favorable conditions for measurement of the activities of these two enzymes [14]. cAMP also increases expression and activity of D1, whereas dexamethasone inhibits D3 mRNA expression and may also inhibit D1 activity [14]. In most cases results were obtained from duplicate or triplicate wells in two independent experiments and the deiodinase activities represent the mean value from the two experiments. In day 28 mouse calvarial osteoblasts D2 activity data were obtained from samples obtained from 3 independent experiments that were pooled and assayed in duplicate because concentrated protein is required for determination of D2 activity to ensure enzyme stability is maximized. D3 activities from these studies were determined in duplicate for each of the 3 independent experiments. Details of the numbers of animals used for tissue and cell preparations, the numbers of independent experiments performed and the numbers of replicate wells assayed per experiment are provided in Tables 1–3.

### Deiodinase activities in cell lysates

ATDC5, MC3T3-E1, SV-HFO and RAW 264.7 cells, and primary chondrocytes, osteoblasts or osteoclasts were washed in ice-cold PBS, sonicated for 20 s in lysis buffer (0.1 M Na^+^ phosphate pH7.2 [0.075 M Na_2_HPO_4_ + 0.028 M NaH_2_PO_4_], 2 mM EDTA, 1 mM DTT) and stored at − 80 °C prior to determination of D1, D2 and D3 enzyme activities. Some proximal tibia growth plates were dissected, excised, minced and sonicated in lysis buffer prior to storage at − 80 °C. D1 assays were performed on undiluted lysates (0.2–0.7 mg/ml) for 120–180 min in the presence of 100 nM rT3 (including 1 × 10^5^ cpm of [3',5'-^125^I]rT3 tracer) and DTT 10 mM at 37 °C, and the absence or presence of 0.1 mM PTU. Adult human liver extracts were included as a positive control for D1 activity in all assays [59]. Reactions were stopped by addition of 0.1 ml ice-cold 5% BSA. Protein-bound [^125^I]iodothyronines were precipitated by addition of 0.5 ml 10% trichloroacetic acid on ice. After centrifugation, supernatants were analyzed for ^125^I^−^ production on Sephadex LH-20 minicolumns (bed volume, 0.25 ml), which were equilibrated and eluted with 0.1 M HCl. D2 assays were performed on undiluted lysates (0.2–0.7 mg/ml) for 120–180 min in the presence of 1 nM T4 (including 1 × 10^5^ cpm of [3',5'-^125^I]T4 tracer) and DTT 10 mM at 37 °C, and the absence or presence of an enzyme saturating concentration of T4 (100 nM or 500 nM). Extracts from COS-1 cells transfected with a human D2 cDNA were included as a positive control in all assays [40]. Release of ^125^I^−^ was determined as described for D1 activity. D3 assays were performed on undiluted lysates (0.2–0.7 mg/ml) for 120 min in the presence of 1 nM T3 (including 2 × 10^5^ cpm of [3'-^125^I]T3 tracer) and DTT 10 mM at 37 °C. Extracts from human endometrial carcinoma ECC1 cells treated with estradiol (10 nM) or human fetal liver were included as positive controls for D3 activity in all assays [37,59]. Reactions were stopped by addition of 0.1 ml ice-cold methanol. After centrifugation, supernatants were mixed with an equal volume of ammonium acetate (pH 4.0), and the mixtures from D1, D2 and D3 assays were analyzed by HPLC as described [29].

### Iodothyronine metabolism in intact cells

ATDC5, SV-HFO and RAW 264.7 cells were cultured with 1 nM (1 × 10^6^ cpm) ^125^I-labeled T4, T3 or rT3 in 0.5 ml medium plus 0.1% BSA for 24 h. After incubation, 100 μl medium was added to 100 μl ice-cold methanol. After centrifugation, 100 μl of supernatant was mixed with 100 μl ammonium acetate (pH 4.0) 0.02 M and 100 μl of the mixture was applied to a 4.6 × 250 mm Symmetry C18 column connected to an Alliance HPLC system (Waters, Etten-Leur, The Netherlands), as described [29]. Radioactivity in the eluate was monitored on line using a Radiomatic A-500 flow scintillation detector (Packard Instruments, Meriden, CT).

### RNA extraction and RT-PCR

MCT8 and D1, D2 and D3 expression was determined by RT-PCR [3,61,70] using the following primers: rat D1 (GenBank NM_021653): forward primer, nucleotides 68–87; reverse, 305–286; rat D2 (NM_031720): forward, 308–327; reverse, 916–896; rat D3 (NM_017210): forward, 99–118; reverse, 447–428; rat glyceraldehyde-3-phosphate dehydrogenase (GAPDH, NM_017008): forward, 597–616; reverse, 1048–1029; mouse D1 (NM_007860): forward, 315–334; reverse, 648–629; mouse D2 (NM_010050): forward, 240–259; reverse, 829–810; mouse D3 (NM_172119): forward, 160–177; reverse, 574–557; mouse MCT8 (AF045692): forward, 552–571; reverse, 933–914; mouse runx-2 (NM_009820): forward, 703–723; reverse, 1084–1064; mouse twist-2 (NM_007855): forward, 151–168; reverse, 698–680; mouse osteocalcin (L24429): forward, 21–40; reverse, 449–429; mouse ALP (X13409): forward, 412–432; reverse, 785–765; mouse collagen Ia1 (X15896): forward, 1247–1268; reverse, 1518–1498; mouse cathepsin K (NM_007802): forward, 54–74; reverse, 663–639; mouse TRAP (NM_007388): forward, 123–145; reverse, 882–861; mouse calcitonin receptor (NM_007588): forward, 376–396; reverse, 1108–1088; mouse GAPDH (NM_008084): forward, 566–585; reverse, 1017–998; mouse 18 S rRNA (X00686) forward, 1577–1596; reverse, 1727–1708. PCRs were performed with a denaturation step at 94 °C for 3 min followed by 30–35 cycles of 30 s at 94 °C, 30 s at 58–64 °C depending on the primer pair, and 30 s at 72 °C, followed by termination at 72 °C for 5 min. PCR products were subcloned and sequenced.

### Quantitative real-time PCR

cDNA was prepared from ATDC5 cells and primary osteoblasts and osteoclasts [74]. Quantitative real-time PCR was performed using an ABI PRISM 7700 system (Applied Biosystems, Nieuwerkerk aan den IJssel, The Netherlands). Reactions were performed in 25 µl Taqman universal PCR master mix (Applied Biosystems), containing 20 ng cDNA. Each reaction contained 200 nM D1 primers (5'-TTAGTTCCATAGCAGATTTTCTTGTCA-3' and 5'-CTGATGTCCATGTTGTTCTTAAAAGC-3') and probe (5'-FAM-AGCCATCTGATGCATGTGCTTCTTCAATG-TAMRA-3'), 200 nM D2 primers (5'-caagtccactcgcggagagt-3' and 5'-gacatgcaccacactggaattg-3') and probe (5'-fam-acgcagcgcagtccctctgagg-tamra-3'), 200 nM D3 primers (5'-TTCCAGAGCCAGCACATCCT-3' and 5'-ACGTCGCGCTGGTACTTAGTG-3') and probe (5'-FAM-TGCACCTGACCACCGTTCATGGC-TAMRA-3') or 300 nM MCT8 primers (5'-CCATAACTCTGTCGGGATCCTC-3' and 5'-ACTCACAATGGGAGAACAGAAGAAG-3') and probe (5'-FAM-ATACCCATCGCGAGGGCTCCGA-TAMRA-3'). mRNA levels were expressed relative to GAPDH. Pre-optimized control GAPDH primers and probe were obtained from Applied Biosystems. PCR reactions were performed for an initial cycle of 2 min at 50 °C and 10 min at 95 °C, followed by 30–35 cycles of 15 s at 95 °C and 1 min at 60 °C. The cycle at threshold (Ct) values represent the cycle numbers at which probe-derived absorbance reached the calculated threshold value. Data are expressed as 2^− ΔCt"ΔCt^ ⁎ 1000 (mRNA copies relative to expression of GAPDH) [45].

### Statistics

Differences between normally distributed data sets were examined for statistical significance using two-tailed Student's *t* tests or by ANOVA followed by Tukey's post-hoc test as appropriate. *P* values of < 0.05 were considered significant.

## Results

### Chondrocytes express MCT8 mRNA

**Fig. 1:**
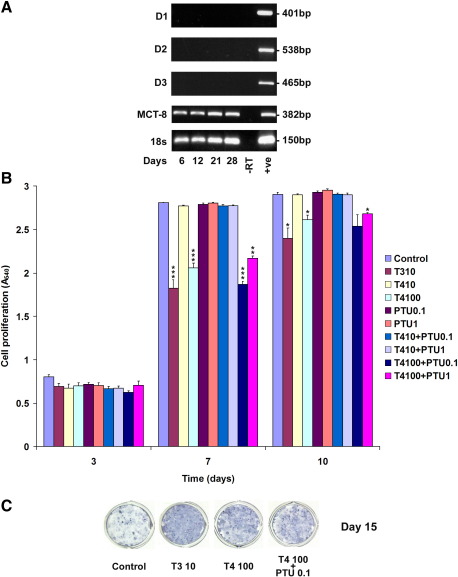
Deiodinase and MCT8 expression and effect of thyroid hormones on chondrogenesis in ATDC5 cells: Panel A shows expression of D1, D2, D3, MCT8 and 18 s ribosomal RNA during ATDC5 cell differentiation (proliferating day 6, cartilage nodule formation day 12, pre-hypertrophic differentiation day 21, hypertrophic differentiation and mineralization day 28) [3,70]. Negative control (− RT) samples in which cDNA template was omitted and positive control (+ ve) samples (liver for D1, heart for D2, brain for D3) are shown. Panel B shows proliferation of ATDC5 cells in response to T3 (10 nM) or T4 (10 nM and 100 nM) in the absence or presence of PTU (0.1 mM and 1 mM) after 3, 7 and 10 days. Data expressed as mean ± SEM, *n* = 6, ⁎*P* < 0.05, ⁎⁎*P*< 0.01’of Figure 1 legend to match occurrence of double asterisk in Panel B of image provided.--> < 0.01, ⁎⁎⁎*P* < 0.001 compared to control. Panel C shows chondrogenesis assessed by alkaline phosphatase staining of cartilage nodules after 15 days in untreated ATDC5 cells and cells treated with T3 10 nM, T4 100 nM and T4 100 nM + PTU 0.1 mM. Images are from single wells of an experiment performed in duplicate and representative of studies performed 3 times.

MCT8 facilitates entry of thyroid hormones into target cells and is a rate-limiting determinant of intra-cellular thyroid hormone metabolism [29]. MCT8 mRNA was expressed at all stages of chondrogenesis in ATDC5 cells cultured over 28 days, whereas D1, D2 and D3 mRNAs were undetectable at all time points (Fig. 1A). In RT-PCR experiments using RNA from 3, 6 and 12 week old rat growth plates, D1 mRNA was also absent. Low levels of D2 and D3 mRNA expression were detectable only following 40 PCR amplification cycles (data not shown). Thus, D1 was not expressed in chondrocytes, whilst D2 and D3 mRNAs were expressed at the limit of detection in growth plate RNA.

### Effects of T4 on chondrogenesis do not involve D1-mediated conversion of T4 to T3

In view of previous studies showing discrepant responses of chondrocytes to thyroid hormones [1,2,16,50,76] effects of T3 and T4 on ATDC5 cell proliferation were examined during the first 10 days of culture, and effects on ALP expression were determined at day 15 (Figs. 1B and C). T3 (10 nM) inhibited cell proliferation, consistent with previous studies [61,70], whereas T4 (10 nM) or the D1-specific inhibitor PTU (0.1 mM and 1 mM) had no effect. T4 (100 nM) also inhibited proliferation but this effect was not blocked by PTU, indicating that a 10-fold higher concentration of T4 than T3 was necessary to inhibit cell growth and that D1-dependent conversion of T4 to T3 was not required (Fig. 1B). T3 (10 nM) and T4 (100 nM) also increased ALP expression and the effect of T4 was not inhibited by PTU, further demonstrating that responses to T4 (100 nM) are independent of D1-mediated conversion of T4 to T3 (Fig. 1C).

### Growth plate chondrocytes only possess D3 enzyme activity

We next determined activities of the D1, D2 and D3 enzymes in chondrocytes and growth plates (Table 1). D1, D2 and D3 activities were undetectable in ATDC5 extracts at all stages of chondrogenesis in 4 independent experiments performed in duplicate. Medium analyzed from ATDC5 cells incubated with ^125^I-labeled T3/T4 for 24 h also revealed no evidence of deiodinase activity following HPLC analysis of iodothyronine metabolites. Furthermore, treatment of ATDC5 cells with dexamethasone, cAMP or TPA did not induce detectable activities of D1, D2 or D3. Similarly, pooled growth plate extracts (*n* = 6–12 per age, 2 experiments in duplicate) from 3, 6 and 12 week old rats lacked detectable D1, D2 or D3 activities. Primary chondrocytes from 6 and 12 week rats (*n* = 6–12 animals per age, 2 experiments in duplicate) also lacked D2 or D3 activity and chondrocytes from 3 week rats had no detectable D2 activity. However, D3 enzyme activity was present in chondrocytes from 3 week rats (*n* = 12 animals per experiment, 2 experiments in duplicate) at levels ranging between 0.6–4.6 fmol/min/mg in control cells and cells treated with dexamethasone, thyroid hormones, dibutyryl cAMP and TPA. No significant induction of D3 activity in response to TPA was evident. D3 activity in control extracts from human fetal liver was 208 fmol/min/mg. Thus, growth plate chondrocytes obtained from weaning rats possessed D3 activity but lacked D1 and D2.

### Osteoblasts express MCT8, D2 and D3 mRNAs throughout differentiation

**Fig. 2:**
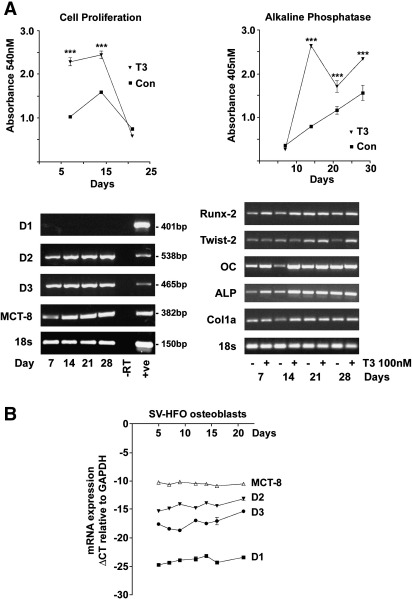
Deiodinase and MCT8 expression in differentiating osteoblasts: Panel A, left graph shows the effect of T3 (100 nM) on proliferation of MC3T3-E1 cells between 7 and 21 days. The graph on the right shows the effect of T3 (100 nM) on alkaline phosphatase activity in MC3T3-E1 cells between 7 and 28 days. Data expressed as mean ± SEM, *n* = 3, ⁎⁎⁎*P* < 0.001 compared to control. RT-PCR gels on left show expression of D1, D2, D3, MCT8 and 18 s ribosomal RNA during mouse calvarial osteoblast differentiation (proliferating day 7, pre-osteoblasts day 14, terminal osteoblasts day 21, mineralization day 28) [10,15,71]. Negative control (− RT) samples in which cDNA template was omitted and positive control (+ ve) samples (liver for D1, heart for D2, brain for D3) are shown. Gels on right show expression of the osteoblast markers Runx-2, Twist-2, osteocalcin (OC), alkaline phosphatase (ALP) and collagen Ia1 (Col1a) in mouse calvarial osteoblasts cultured for 28 days in the absence or presence of T3 (100 nM). Panel B shows real-time qRT-PCR analysis of D1, D2, D3 and MCT8 expression in differentiating human SV-HFO cells. ΔCT indicates cycle threshold detection level of MCT8 or deiodinase expression relative to GAPDH; data expressed as mean ± SEM, *n* = 3 each time point.

The osteoblastic phenotype of MC3T3 cells in the absence and presence of T3 was confirmed in RT-PCR studies, which revealed expression of the osteoblast marker genes Runx-2, Twist-2, osteocalcin, alkaline phosphatase and type 1 collagen throughout the 28 day cell culture period (Fig. 2A). T3 (1–100 nM) stimulated MC3T3-E1 cell proliferation after 7 and 14 days, and increased ALP activity after 7, 14, 21 and 28 days (Fig. 2A, 100 nM response shown), demonstrating that T3 induces differentiation of MC3T3-E1 cells, which undergo a defined programme of osteoblastogenesis [48]. MCT8, D2 and D3 mRNAs were expressed in primary mouse osteoblasts at all stages of differentiation whereas D1 was undetectable (Fig. 2A). Similar findings were observed in MC3T3-E1 cells (data not shown). To investigate whether progression of osteoblast differentiation was associated with altered expression of MCT8 and deiodinases, we examined their expression by qRT-PCR in human fetal osteoblastic SV-HFO cells, which differentiate over a period of 21 days [20,25]. MCT8 was expressed throughout the culture period with a cycle detection threshold (ΔC_T_) relative to GAPDH of between − 10 to − 11 cycles (ΔC_T_ = C_T GAPDH_ − C_T MCT8_). D2 and D3 mRNAs were expressed at lower levels than MCT8 (ΔC_T_ for D2 = − 15 to − 13, ΔC_T_ for D3 = − 18 to − 15), whereas expression of D1 was at the limit of detection (ΔC_T_ = − 23 to − 25) (Fig. 2B). Thus, mouse and human osteoblasts expressed D2 and D3 mRNAs throughout differentiation but did not express a significant amount of D1.

### Osteoblasts possess D3 enzyme activity throughout differentiation and D2 activity is present in mature osteoblasts

Extracts from differentiated MC3T3-E1 cells cultured for 28 days did not possess D1, D2 or D3 enzyme activities (*n* = 2–3 experiments in duplicate) (Table 2). Furthermore, treatment of MC3T3-E1 cells with dexamethasone, cAMP or TPA did not induce detectable activities of D1, D2 or D3. Rat and mouse calvarial osteoblasts also undergo a defined programme of differentiation over 28 days [10,71]. Similar to MC3T3-E1 cells, extracts from mature differentiated primary mouse osteoblasts did not possess detectable D1 activity (*n* = 3) but, by contrast, expressed specific D2 (0.21 fmol/min/mg) and D3 (1.35 fmol/min/mg) activities (Table 2). Activities of D1, D2 and D3 were also determined in rat calvarial osteoblasts cultured for 11, 18, 20 and 27 days in the absence or presence of T3, T4 or dexamethasone (*n* = 2 experiments in duplicate). Rat osteoblasts did not express detectable D1 or D2 activities but possessed specific D3 activity in the absence and presence of T3, T4 or dexamethasone at all time points (Table 2). No alteration of D3 activity in response to these agents was evident. Extracts from T3, T4 and dexamethasone-treated human SV-HFO cells at days 1, 7, 14 and 21 did not possess D1 or D2 activities (Table 2), and medium analyzed from cells incubated with ^125^I-labeled T3/T4 for 24 h prior to harvest at each time point also revealed no evidence of D1 or D2 activities following HPLC analysis (*n* = 2 in duplicate). Treatment of SV-HFO cells with dexamethasone, cAMP or TPA did not induce detectable D1 or D2 activity. D3 activity was also undetectable in T3 and T4 treated SV-HFO cells at each time point, whereas low levels of D3 activity were present in dexamethasone-treated cells at days 14 and 21. Thus, primary osteoblasts possessed D3 enzyme activity throughout differentiation and mature mouse osteoblasts also expressed D2 activity.

### Osteoclasts express varying levels of MCT8, D2 and D3 mRNAs during differentiation

**Fig. 3:**
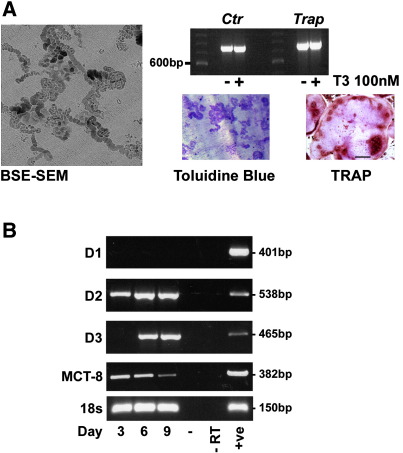
Deiodinase and MCT8 expression in differentiating osteoclasts: Panel A shows formation of resorption pit trails on dentine slices by differentiating osteoclasts cultured with RANKL for 9 days and imaged by back-scattered electron scanning electron microscopy (BSE-SEM) or by light microscopy following staining with toluidine blue [6]. Multinucleated osteoclasts were visualized following immunohistochemical staining for TRAP [6], and differentiated cells expressed the calcitonin receptor (*Ctr*) and *Trap* mRNAs. Panel B shows expression of D1, D2, D3, MCT8 and 18 s ribosomal RNA during osteoclastogenesis in bone marrow osteoclasts cultured in the presence of RANKL for 3–9 days. Negative control (− RT) samples in which cDNA template was omitted and positive control (+ ve) samples (liver for D1, heart for D2, brain for D3) are shown.

Culture of bone marrow from 6–8 week old rats or mice in the presence of RANKL for 9 days resulted in the generation of multinucleated osteoclasts that resorbed dentine and expressed the calcitonin receptor, TRAP (Fig. 3A) and cathepsin K (not shown). Differentiating osteoclasts expressed MCT8, D2 and D3 mRNAs but not D1 mRNA. D2 was expressed throughout osteoclastogenesis, whereas D3 was absent during the first 3 days of culture but expressed later at days 6 and 9. MCT8 expression declined as osteoclastogenesis progressed (Fig. 3B). Thus, osteoclasts expressed varying levels of D2 and D3 mRNAs during differentiation but did not express D1 mRNA.

### Osteoclasts only possess D3 enzyme activity

Bone marrow cultures from 6–8 week old animals did not possess detectable D1 or D2 activity in cell extracts, whereas D3 activity was present in monocytic cells in the absence of RANKL and in osteoclasts differentiated in the presence of RANKL (*n* = 2 experiments in triplicate, Table 3). RAW 264.7 cells undergo osteoclast differentiation in response to RANKL over a period of 2–5 days [43]. Untreated and RANKL treated cells after 3, 5 and 7 days in culture did not possess detectable D1, D2 or D3 enzyme activity in cell extracts (*n* = 2 experiments in triplicate). Furthermore, medium from RAW 264.7 cells incubated with ^125^I-labeled T3/T4 for 24 h prior to harvest at each time point revealed no evidence of deiodinase activity following HPLC analysis (Table 3). Thus, primary bone marrow osteoclasts expressed D3 enzyme activity.

## Discussion

There are incomplete and conflicting reports regarding the functional expression of the iodothyronine deiodinase enzymes in skeletal cells [1,17,24,30,42,49–51,69,76] and their physiological importance in bone has not been explored*in vivo*. Here, we show that D1 is not expressed in skeletal cells, D2 activity is restricted to mature osteoblasts, and functional D3 enzyme is present in primary chondrocytes, osteoblasts and osteoclasts.

In these studies, we show that D1 mRNA expression and enzyme activity are undetectable in chondrocytes, osteoblasts and osteoclasts from several sources, thus extending previous work in which D1 activity was not detected in osteoblastic cell lines [24,30,42]. These findings are in agreement with general observations in inbred D1-deficient C3H/HeJ mice, which exhibit minor disturbances of the HPT axis with normal T3 and TSH levels and modestly elevated T4 levels, but have normal growth [11,67]. Furthermore, D1 knockout (D1^−/−^) mice have a similar mild disturbance of the HPT axis and also display normal growth with no obvious skeletal defect reported [66]. We conclude, therefore, that D1 does not have a physiological role in the skeleton.

To investigate D2 activity, we employed a highly specific and sensitive D2 assay based on the measurement of radiolabeled T3 production as determined by HPLC [29]. This approach contrasts with previous studies [50,51], which relied solely on sensitive detection of ^125^I^−^ release that can result from non-specific substrate degradation [30]. Our results unequivocally demonstrate the presence of specific D2 activity in mature mouse calvarial osteoblasts, whereas specific D2 activity was undetectable in all other cells studied.

There are no previous data in osteoclasts but the lack of specific D2 activity in chondrocytes is apparently at odds with a previous study of ATDC5 cells and growth plate organ cultures, which concluded that deiodination of labeled rT3 in the presence of PTU was due to D2-like activity [50]. This study, however, has been criticized as being non-specific [30] because deiodination was only detected after prolonged assay incubation and its kinetics, response to thyroid hormones and proteasome inhibition were not assessed. Substantial PTU-insensitive, non-saturable and non-specific outer ring deiodination has been identified previously in bone cells [30] and this is likely be the source of deiodination observed by Miura et al. [50]. Previous studies in SaOS2 and NHOst osteoblastic cells have also reported substantial levels (5–6 fmol/mg/min) of 5'-deiodination [51]. However, detection of D2-like activity in osteoblastic cells, similar to studies in ATDC5 cells and growth plate organ cultures [50], relied on the measurement of ^125^I^−^ released from cell extracts [51]. It seems reasonable, therefore, to conclude that levels of specific D2 activity contributing to the total 5'-deiodination observed by Morimura in osteoblastic cells may be much lower than 5-6 fmol/mg/min. Indeed, the 0.21 fmol/min/mg specific D2 activity determined in mature primary murine osteoblasts in the current studies is in close agreement with the levels of well-characterized specific D2 activity (0.2–0.3 fmol/min/mg) observed previously in mouse femur and bone marrow [30]. Nevertheless, in contrast to the current study, Gouveia et al. [30] also demonstrated specific D2 activity in differentiated confluent MC3T3-E1 cells in the presence of high Se concentrations. The discrepancy between these findings and the current studies may reflect different cell culture conditions or clonal differences between batches of MC3T3-E1 cells.

Even though specific D2 activity was only present in differentiated mature murine osteoblasts, D2 mRNA was detected more widely in growth plate chondrocytes (after 40 PCR cycles), primary rat osteoblasts, MC3T3 and SV-HFO osteoblastic cells and osteoclasts. Discordance between D2 mRNA expression and enzyme activity has been documented before in skeletal cells. For example, Dentice et al. showed specific expression of D2 mRNA in hypertrophic chondrocytes in the developing 8 day old chick, but did not detect enzyme activity [23]. The D2 assay used in the current studies is highly specific and sensitive [29], and enzyme activity was investigated in cell and tissue extracts and in intact cells. Although it is possible that enzyme activity was below the level of detection in all cells other than mature murine osteoblasts, we conclude that if this was the case the specific D2 activity must be so low that it is reasonable to propose D2 does not have such an important physiological role in bone as postulated previously. An alternative possibility is that cells expressing D2 mRNA encode a non-functional deiodinase. A more likely and attractive explanation is that D2 activity is eliminated efficiently in bone cells following degradation of the enzyme. The D2 protein is labile with only a 20-min half-life because of post-translational regulation by selective ubiquitination and targeted proteasomal degradation [13,78]. Indeed, ubiquitin-mediated degradation of D2 is known to regulate thyroid hormone activation in the embryonic growth plate [23] and other tissues [13,26,63] thereby accounting for discordance between D2 mRNA expression and enzyme activity.

Limited information is available regarding the effect of D2-deficiency on the skeleton *in vivo*. D2^−/−^ mice have pituitary resistance to T4 with normal circulating T3 concentrations, but 1.4-fold elevated T4 and 2-fold elevated TSH levels in adults. A transient period of 9% reduced weight was evident between 4 and 7 weeks of age in males but not females [65]. Recently, C3H/HeJ D2^−/−^ compound mutant mice with D1 deficiency and deletion of D2 were shown to maintain normal growth and have normal T3 levels [21]. In agreement with the current studies documenting an absence of D1 and D2 activities in growth plate extracts and chondrocytes, findings in D1 and D2 mutant mice indicate the supply of T3 to the skeleton during growth is not compromised in the absence of either or both enzymes. Together with findings in primary osteoclasts and RAW 264.7 cells, we conclude D2 does not have a physiological role in growth plate chondrocytes or osteoclasts. Findings in differentiated murine primary osteoblasts however suggest a possible role for D2 in mature osteoblast function. To investigate further it will be necessary to examine the consequences of D2 deletion in the adult skeleton, as osteoblast D2 may regulate adult bone mineralization.

In contrast to the lack of D1 and restricted presence of D2 in bone, D3 activity was more widespread. Specific D3 activity was present in growth plate chondrocytes cultured from weaning 3 week old rats and in all primary osteoblasts, primary monocytes and primary osteoclasts examined. By contrast, D3 activity was absent from ATDC5, MC3T3, SV-HFO and RAW 264.7 cell lines, indicating that immortalized and transformed cell lines do not adequately represent the D3-expressing phenotype of primary bone cells. Interestingly, D3 activity was present only in chondrocytes from 3 week old rats but not in cells from older animals. The lack of D3 activity in cells from 6 and 12 week animals, suggests that the enzyme may limit availability of thyroid hormones in the growth plate until after weaning when T3-dependent linear growth acceleration is initiated [7,32]. D3 activity was also expressed in differentiating primary osteoblasts and osteoclasts and we conclude, therefore, that that local T3 availability in bone cells is controlled principally via D3-dependent regulation of thyroid hormone catabolism.

Interestingly, D3^−/−^ mice have neonatal thyrotoxicosis with a 4-fold elevated T3 at post-natal day 5 due to diminished T3 clearance that is followed by persistent central hypothyroidism from 2 weeks of age. Severe growth retardation at weaning persists into adulthood and remains at 1 year of age [33,34]. We showed previously that mice with skeletal thyrotoxicosis resulting from mutation of TRβ display short stature due to accelerated bone maturation *in utero* and premature growth plate quiescence in the post-weaning period [4,5,57]. Furthermore, TRα mutant mice with skeletal hypothyroidism are growth retarded in the post-weaning period because of delayed endochondral ossification [5,55,56]. The short stature observed in D3^−/−^ mice may therefore reflect accelerated bone maturation due to excessive exposure to thyroid hormones prior to weaning because of absent D3 expression in bone during this critical period. Additionally, systemic hypothyroidism after 2 weeks of age will further delay growth in the post-weaning period when systemic thyroid hormones are essential, resulting in the severe growth phenotype observed in D3^−/−^ mice [33,34]. We hypothesize, therefore, that D3 plays an essential role to limit thyroid hormone availability in the immature skeleton and regulate bone development and linear growth. The role of D3 in adult bone is difficult to predict and analysis of the skeleton in D3^−/−^ mice is likely to be informative.

In conclusion, these studies suggest that thyroid hormone access and availability to bone is unlikely to be a limiting factor that regulates thyroid hormone responsiveness in the developing skeleton. Indeed, we show that MCT8 mRNA is expressed in all bone cell lineages. Rather, the developing skeleton may be unusual as it appears that control of thyroid hormone availability is determined by D3-mediated catabolism. This contrasts with other tissues such as cochlea and brain in which hormone availability is determined mainly by D2 [31,54], although D3 also plays a role in fetal brain by regulating the spatial availability of thyroid hormones [38]. In mature osteoblasts hormone availability is likely to be tightly regulated at the level of both T3 production and catabolism by the relative activities of D2 and D3. The current findings provide new insight into our understanding of thyroid hormone action in skeletal cells but our understanding of the physiological role of the deiodinase enzymes in bone *in vivo* will require detailed analysis of deiodinase mutant mice.

## Figures and Tables

**Table 1:** Deiodinase enzyme activities in growth plate tissue extracts and chondrocytes

	ATDC5 2, 7, 14, 21 day extracts and intact cells *n* = 4 in duplicate	Growth plate tissue 3, 6, 12 week rat extracts 6–12 rats/age/experiment *n* = 2 in duplicate	1° chondrocytes 3 week rat extracts 12 rat s/experiment *n* = 2 in duplicate	1° chondrocytes 6, 12 week rat extracts 6 rats/age/experiment *n* = 2 in duplicate
D1 (fmol/min/mg)	0	0	N/D	N/D

D2 (fmol/min/mg)	0	0	0	0

D3 (fmol/min/mg)				
Con	0	0	2.9	0
Dex 1 μM	0	0	1.2	0
T3 100 nM	0	0	0.6	0
T4 100 nM	0	0	1.9	0
cAMP 1 mM	0	0	4.6	0
TPA 0.1 μM	0	0	4.3	0

D1 enzyme activities were not determined in samples from primary chondrocytes because of limiting amounts of cell extract. Positive control adult human liver extracts deiodinated 100% of substrate in each 120 min D1 assay; COS-1 cell extracts transfected with human D2 possessed 12.06 fmol/min/mg D2 activity; ECC1 cell and human fetal liver extracts possessed D3 activities of 9.83 and 208 fmol/min/mg, respectively.

**Table 2:** Deiodinase enzyme activities in osteoblasts

	MC3T3 day 28 extracts *n* = 2–3 in duplicate	Mouse calvarial day 28 extracts 6–8 mice *n* = 3 in duplicate	Rat calvarial day 11 extracts 6–8 rats *n* = 2 in duplicate	Rat calvarial day 18 extracts 6–8 rats *n* = 2 in duplicate	Rat calvarial day 20 extracts 6–8 rats *n* = 2 in duplicate	Rat calvarial day 27 extracts 6–8 rats *n* = 2 in duplicate	SV-HFO 1, 7, 14, 21 day extracts and intact cells *n* = 2 in duplicate
D1 (fmol/min/mg)	0	0	0	0	0	0	0

D2 (fmol/min/mg)	0	0.21 *n* = 3 pooled in duplicate	0	0	0	0	0

D3 (fmol/min/mg)							
Con	0	1.35 ± 0.15	1.5	0.4	2.5	3.1	0
Dex 1 μM	0	N/D	1.9	1.8	6.0	4.3	0.5 d14
							2.5 d21
T3 100 nM	0	N/D	2.0	2.7	2.5	3.5	0
T4 100 nM	0	N/D	1.4	3.4	2.8	3.5	0

Positive control adult human liver extracts deiodinated 100% of substrate in each 120 min D1 assay; COS-1 cell extracts transfected with human D2 possessed 12.06 fmol/min/mg D2 activity; ECC1 cell and human fetal liver extracts possessed D3 activities of 9.83 and 208 fmol/min/mg, respectively.

**Table 3:** Deiodinase enzyme activities in osteoclasts

	Primary rat monocyte − RANKL extracts 3 rats *n* = 2 in triplicate	Primary rat osteoclast + RANKL extracts 3 rats *n* = 2 in triplicate	RAW 264.7 3, 5, 7 day extracts and intact cells *n* = 2 in triplicate
D1 (fmol/min/mg)	0	0	0
D2 (fmol/min/mg)	0	0	0
D3 (fmol/min/mg)	1.13	1.0	0

Positive control adult human liver extracts deiodinated 100% of substrate in each 120 min D1 assay; COS-1 cell extracts transfected with human D2 possessed 12.06 fmol/min/mg D2 activity; ECC1 cell and human fetal liver extracts possessed D3 activities of 9.83 and 208 fmol/min/mg, respectively.
